# Harnessing HLA Divergence for Improved Donor Selection in Haploidentical Haematopoietic Stem Cell Transplantation

**DOI:** 10.1111/tan.70491

**Published:** 2025-12-10

**Authors:** Debora Jorge Cordeiro, Juliette Villemonteix, Alienor Xhaard, Ophélie Ferrary, William Dartois, Bruno A. Lima, Laurie Toullec, Regis Peffault de Latour, Sophie Caillat‐Zucman, Vincent Allain

**Affiliations:** ^1^ Laboratoire d'Immunologie et Histocompatibilité, Hôpital Saint‐Louis, Assistance Publique‐Hôpitaux de Paris (AP‐HP) Université Paris Cité Paris France; ^2^ Service d'Hématologie‐Greffe Hôpital Saint‐Louis, AP‐HP Paris France; ^3^ Oficina de Biostatistica Ermesinde Portugal; ^4^ INSERM UMR1342, Institut de Recherche St‐Louis Université Paris Cité Paris France

**Keywords:** haematopoietic stem cell transplantation (HSCT), haploidentical donor, HLA evolutionary divergence (HED), immunopeptidome

## Abstract

Haploidentical haematopoietic stem cell transplantation (haplo‐HSCT) with post‐transplant cyclophosphamide (PTCy)‐based graft‐versus‐host disease (GVHD) prophylaxis is commonly used in patients without a fully matched donor. HLA evolutionary divergence (HED), a surrogate for the diversity of the immunopeptidome, can predict HLA‐matched HSCT outcomes. Because of the peculiarities of HED measurement in haplo‐HSCT, we designed a new HED_D‐>R_ metric to proxy the fraction of the recipient‐specific immunopeptidome not recognised as self by the haplo‐identical donor, and evaluated its predictive value on haplo‐HSCT outcomes with the aim of providing help in choosing the best donor. We retrospectively studied 104 patients who underwent PTCy haplo‐HSCT for haematological malignancy and calculated per‐locus and per‐class HED in the recipient (HED_R_), the donor (HED_D_), across mismatched haplotypes (HED_MM_) as well as HED_D‐>R_. Patients with low class 1 HED_R_ had worse OS (hazard ratio (HR) 2.16, 95% confidence intervals (CI) 1.06–4.40, *p* = 0.033), whereas no significant impact of HED_D_ or HED_MM_ at any HLA locus was observed. Moreover, in multivariate analysis of patients surviving at least 100 days, low class 1 HED_D‐>R_ was associated with inferior OS (HR 2.67, 95% CI 1.13–6.28; *p* = 0.025). In addition to strengthening the impact of recipient class 1 HED on HSCT outcomes regardless of donor type, our results suggest that our novel, publicly available, HED_D‐>R_ metric could help select the most appropriate donor among haploidentical candidates.

## 1 Introduction

Haploidentical haematopoietic stem cell transplantation (haplo‐HSCT) with post‐transplant cyclophosphamide (PTCy)‐based graft‐versus‐host disease (GVHD) prophylaxis is widely used in patients with haematological malignancies who lack a fully matched donor. Currently, the choice of the optimal donor among haploidentical candidates (biological parents, children and siblings) is mainly based on non‐HLA factors such as age, gender, donor‐specific anti‐HLA antibodies and CMV status [1]. However, although they are not yet used routinely in clinical practice, some germline HLA characteristics may help better predict haplo‐HSCT outcomes [2, 3].

HLA evolutionary divergence (HED) is a quantifiable measure of the physicochemical differences between the peptide‐binding domains of the two HLA alleles at each locus. As demonstrated for pathogen‐derived peptides, HED is a relevant surrogate for the diversity of the peptide repertoire (immunopeptidome) presented by HLA molecules and therefore, the strength of the T‐cell response [4, 5, 6, 7]. Accordingly, HED may reflect the ability of HLA molecules to present tumour‐associated antigens or alloantigens to T cells mediating the graft‐versus‐tumour (GvT) and GVHD effects in the context of allogeneic HSCT. Indeed, HED appears to be a useful tool to predict the response to cancer immunotherapy [8, 9], solid organ transplant rejection [10] and outcomes of HLA‐matched HSCT [11, 12, 13, 14]. Consistent with this notion, recent works demonstrated that immunopeptidome divergence can be predicted based on differences in peptide binding motifs (PBM), which are experimentally determined by the biochemical characteristics of the peptides that an allele can present. Using PBM groups as a proxy for immunopeptidome divergence between mismatched HLA alleles, recent works have shown that differences in the PBM of mismatched HLA alleles may also inform clinical outcomes in unrelated HSCT [15, 16, 17, 18]. Altogether, these findings provide compelling evidence that immunopeptidome divergence between mismatched HLA molecules may serve as a biomarker for risk stratification in unrelated HSCT.

Assessing the impact of HED in the haplo‐HSCT setting is more complex, as it must take into account the divergence between the two alleles of the recipient, the two alleles of the donor and the mismatched haplotypes. Moreover, it should be considered that donor‐derived T cells will recognise as self a fraction of the recipient's immunopeptidome presented by HLA molecules of the shared haplotype. Indeed, since a haplotype is shared by the donor and the recipient, a fraction of the recipient's immunopeptidome is also presented by the donor's HLA molecules, and will therefore be recognised as self by the donor‐derived T cells. To account for this peculiarity, we leveraged the HED metric to capture the diversity of the recipient‐specific, that is, non‐donor‐shared, immunopeptidome (HED_D‐>R_), assuming that it would proxy donor‐versus‐recipient alloreactivity and evaluated its predictive value on haplo‐HSCT outcomes.

## 2 Patients and Methods

We retrospectively studied 104 patients who underwent haplo‐HSCT with PTCy for haematological malignancy between 2014 and 2022 at Saint‐Louis hospital. HLA‐A, ‐B, ‐C, ‐DRB1, ‐DQB1 and ‐DPB1 typing at second field (four‐digit) resolution and clinical data were available for all patients and donors. HED was calculated as the genetic distance between allele pairs at each HLA locus in the recipient (HED_R_), the donor (HED_D_) or across mismatched haplotypes (HED_MM_) using the Grantham distance [4]. Class 1 HED represents the arithmetic mean of HLA‐A, ‐B and ‐C HED values and class 2 HED the arithmetic mean of HLA‐DRB1, ‐DQB1 and ‐DPB1 HEDs. Since HEDs are not normally distributed (Figure S1), high and low HEDs were categorised as being above or below the first quartile value in the entire cohort.

**FIGURE 1:**
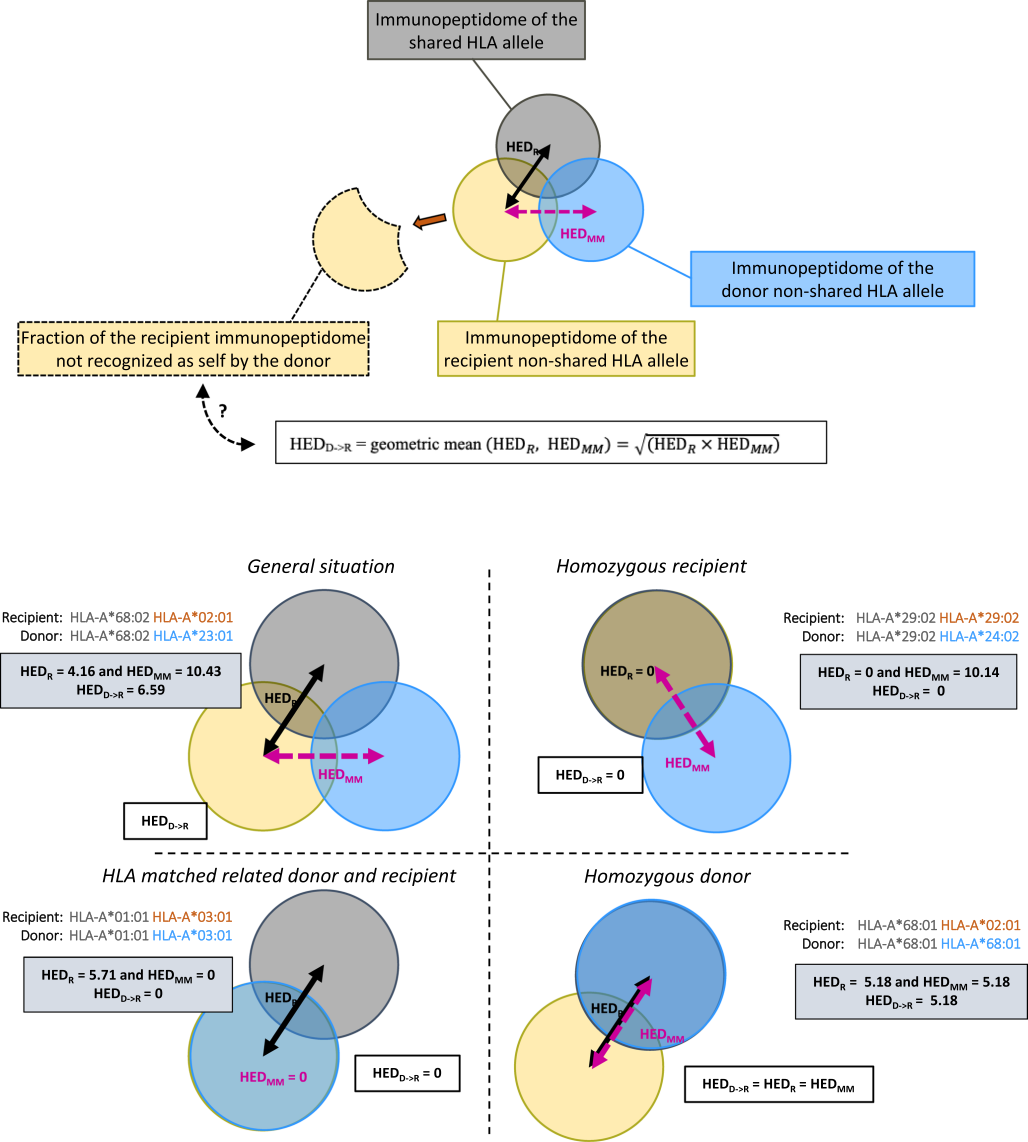
Design of the HED_D‐>R_ metric. Upper panel: HED_D‐>R_ is determined for each HLA locus as the geometric mean $\left({\mathrm{HED}}_{R},\;{\mathrm{HED}}_{\mathrm{MM}}\right)=\sqrt{\left({\mathrm{HED}}_{R}\;\mathrm{at}\;a\;\text{given locus}\times {\mathrm{HED}}_{\mathrm{MM}}\;\mathrm{at}\;\text{this locus}\right)}$. Lower panel: Representative examples with HLA typing data of the recipient and the donor, and the corresponding HED_R_, HED_MM_ and HED_D‐>R_ values.

We designed a novel HED_D‐>R_ metric that integrates both HED_R_ and HED_MM_ to estimate the portion of the recipient immunopeptidome that is not shared with the donor and may therefore become a target of alloreactivity, as illustrated in Figure 1. The three following specifications were applied in the definition of HED_D‐>R_. First, HED_D‐>R_ had to be a monotonically increasing function of both HED_MM_ and HED_R_. As shown in Figure 1, we hypothesised that higher HED_MM_ and HED_R_ values would correspond to a lower portion of shared immunopeptidome between the donor and the recipient (and therefore, a higher proportion of the recipient's immunopeptidome that could become a target of alloreactivity). Second, for a given locus, we required that HED_D‐>R_ = 0 if the recipient was homozygous (HED_R_ = 0) or if the recipient and donor alleles were identical (HED_MM_ = 0). In these situations, we indeed hypothesised that the recipient's immunopeptidome would be included in the donor's immunopeptidome and therefore recognised as self. Third, if the donor was homozygous at a given locus, HED_R_ = HED_MM_ for this locus (by definition), and we imposed HED_D‐>R_ = HED_R_ = HED_MM_.

Based on these different criteria, we defined HED_D‐>R_ for each HLA locus as the geometric mean of $\left({\mathrm{HED}}_{R},\;{\mathrm{HED}}_{\mathrm{MM}}\right)=\sqrt{\left({\mathrm{HED}}_{R}\;\mathrm{at}\;a\;\text{given locus}\times {\mathrm{HED}}_{\mathrm{MM}}\;\mathrm{at}\;\text{this locus}\right)}$. Class 1 HED_D‐>R_ was calculated as the arithmetic mean of HED_D‐>R_ at HLA‐A, ‐B and ‐C, and Class 2 HED_D‐>R_ was calculated as the arithmetic mean of HED_D‐>R_ at HLA‐DRB1, ‐DQB1 and ‐DPB1.

Reported statistics are medians (interquartile range, IQR) or numbers and percentages according to the type of variable. Overall survival (OS) and disease‐free survival (DFS) were estimated by the Kaplan–Meier method and groups were compared with the log‐rank test. The cumulative incidence of relapse (CIR) was estimated using non‐relapse mortality (NRM) as a competing risk. The cumulative incidence of grade 2–4 acute GVHD (aGVHD) at 100 days was estimated with death or relapse as competing risks. Measurement of prognostic factors associations used hazard ratio (HR) with 95% confidence intervals (CI), computed from univariable, then multivariable Cox models for OS and DFS, and Fine‐Gray sub‐distribution hazard regression for CIR and GVHD incidence. All patients gave their consent for data collection before transplantation.

## 3 Results

The main characteristics of the 104 recipient/donor pairs are reported in Table 1. All patients and donors had available high‐resolution HLA genotyping (Data Source S1). Median follow‐up time was 668 days (IQR 343–1365). Three‐year probabilities of OS, DFS, NRM and CIR were 57.6%, 56%, 12% and 30.4%, respectively. The cumulative incidence of grade 2–4 aGVHD at day 100 was 53.5%. No specific patient‐, disease‐, or transplant‐related characteristic was significantly associated with HSCT outcomes, except for a lower survival and a higher risk of relapse in patients with a higher disease risk index (DRI) (*p* = 0.026 and 0.020, respectively). In particular, donor and recipient age, female donor to male recipient combination, donor‐recipient kinship, donor/recipient CMV serostatus and HLA mismatch did not significantly affect transplant outcomes.

**TABLE 1:** Patient characteristics.

		All patients (*n* = 104)
Age at HSCT (years)	Recipient	46 [29–59]
	Donor	45.5 [27–45]
Gender	Male	63 (60.6)
	Female	41 (39.4)
Female donor to male recipient		24 (23.1)
Diagnosis	AML	47 (45.2)
	ALL	15 (14.4)
	MDS	18 (17.3)
	MPN	9 (8.7)
	Lymphoma	13 (12.5)
	Bone marrow failure	2 (1.9)
DRI	High	37 (35.6)
	Intermediate	61 (58.7)
	Low	4 (3.8)
	Non applicable	2 (1.9)
HSCT number	First	97 (93.3)
	Second	7 (6.7)
Stem cell source	Bone marrow	49 (47.1)
	Peripheral blood	55 (52.9)
Donor/recipient kinship	Parent	30 (28.8)
	Child	34 (32.7)
	Sibling	38 (36.5)
	Other	2 (1.9)
Conditioning	MAC	38 (36.5)
	RIC	66 (63.5)
TBI	Yes	25 (24)
	No	79 (76)
R/D CMV serostatus	Neg/neg	21 (20.2)
	Pos/neg	12 (11.5)
	Neg/pos	4 (3.8)
	Pos/pos	67 (64.4)

*Note:* Continuous variables are expressed as median [IQR], and categorical variables as number (%) of individuals.

Abbreviations: ALL, acute lymphoid leukaemia; AML, acute myeloid leukaemia; CMV, cytomegalovirus; D, donor; DRI, disease risk index; HSCT, haematopoietic stem cell transplantation; MAC, myeloablative conditioning; MDS, myelodysplastic syndrome; MPN, myeloproliferative neoplasm; Neg, negative; Pos, positive; R, recipient; RIC, reduced intensity conditioning; TBI, total body irradiation.

**FIGURE 2:**
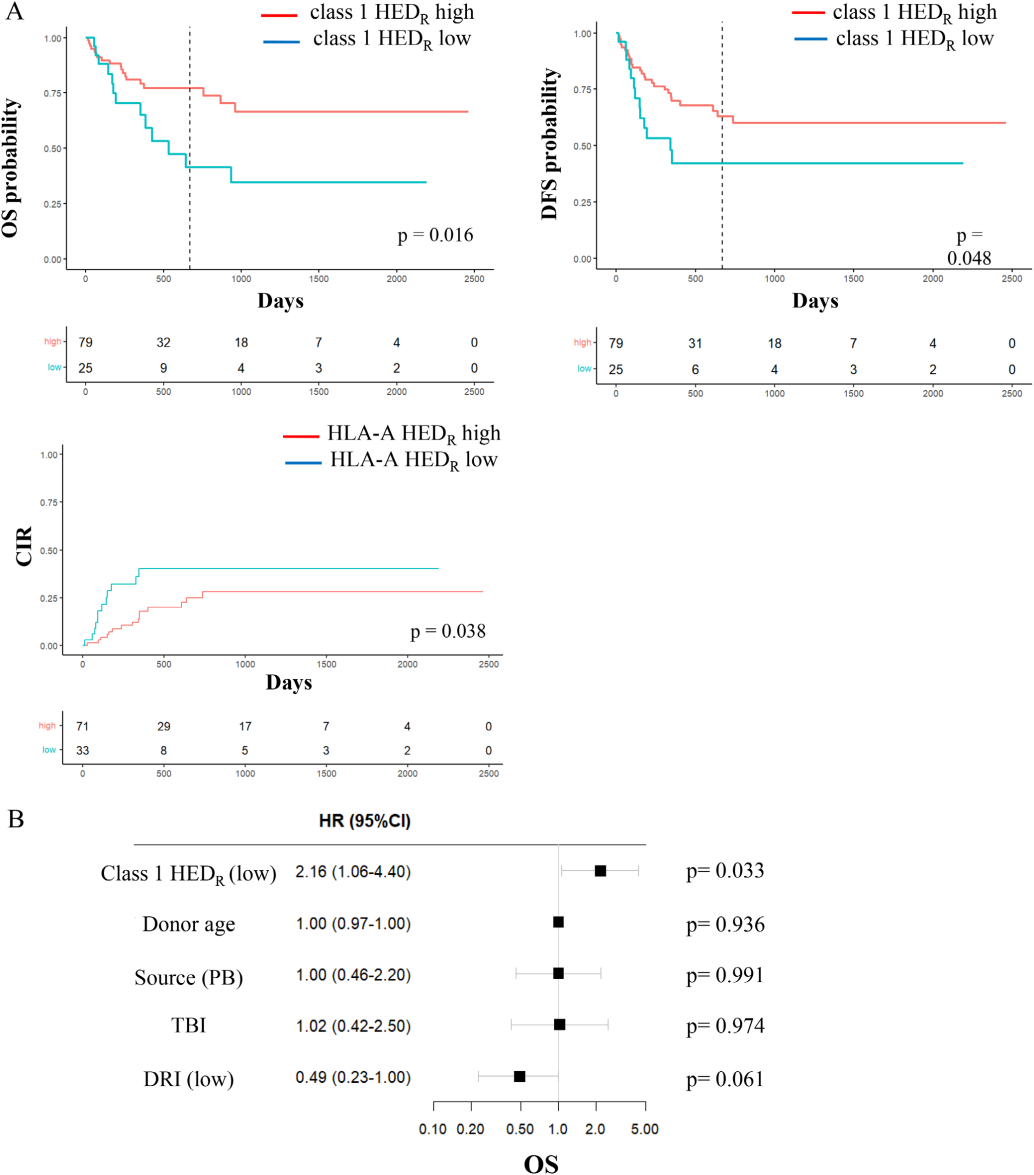
Survival estimates based on HED_R_ values. (A) Univariable Kaplan Meier estimates of overall survival (OS) and disease‐free survival (DFS) and cumulative incidence of relapse (CIR) based on class 1 HED_R_ values above or below the first quartile. Non adjusted *p*‐values indicate the significance of the log‐rank test. Vertical dotted line indicates the median follow‐up time. (B) Multivariable cox regression analysis of OS including class 1 HED_R_ as main effect variable.

For each patient, we calculated per‐locus and per‐class HED values (Table 2) and evaluated the impact of HED on standard HSCT outcomes (Table 3). Low class 1 HED_R_ was significantly associated with reduced OS (*p* = 0.016) and DFS (*p* = 0.048) (Figure 2A). This effect remained significant in the 62 patients fully heterozygous at HLA class 1 loci (*p* = 0.008 and *p* = 0.028, respectively). No significant associations were observed for relapse, NRM or aGVHD. In multivariate analysis including donor age, stem cell source, total body irradiation‐based conditioning and DRI, selected as relevant covariables based on our clinical practice, patients with low class 1 HED_R_ had worse OS (HR 2.16, 95% CI 1.06–4.40; *p* = 0.033) (Figure 2B). Therefore, class 1 HED_R_ informs survival probability after haplo‐HSCT, in line with previous observations in HLA‐matched HSCT [11, 12, 13], adding further evidence of the value of this metric in informing HSCT outcomes regardless of donor type. However, this information does not provide any help in choosing the best haploidentical donor, which was the premise of the study.

**TABLE 2:** Distribution of HED values.

Variable	Median	Lower quartile	Upper quartile	Minimum	Maximum
HED_R_					
HLA class I	6.5	5.18	7.38	0	10.1
HLA‐A	6.98	4.45	9.47	0	12.97
HLA‐B	7.41	6.01	9	0	13.34
HLA‐C	5.39	3.37	6.85	0	7.93
HLA class II	8.83	6.15	10.26	0	15.37
HLA‐DRB1	8.82	4.82	13.72	0	19.13
HLA‐DQB1	10.96	5.12	14.67	0	19.2
HLA‐DPB1	4.28	0.64	7.52	0	10.56
HED_D_					
HLA class I	6.75	4.87	7.74	0.32	10.31
HLA‐A	6.43	3.75	9.04	0	12.97
HLA‐B	7.72	6.44	9.74	0.37	14.25
HLA‐C	5.51	4.44	6.84	0	8.16
HLA class II	8.83	6.84	10.76	0	14.97
HLA‐DRB1	10.72	7.78	13.26	0	19.13
HLA‐DQB1	11.11	7.02	16.29	0	19.2
HLA‐DPB1	4.01	0.64	6.87	0	10.32
HED_MM_					
HLA class I	6.46	5.52	7.64	0	10.8
HLA‐A	6.96	4.35	10.21	0	12.97
HLA‐B	8.02	5.79	10.35	0	14.25
HLA‐C	5.01	3.46	6.77	0	8.16
HLA class II	8.2	5.94	10.82	0	15.01
HLA‐DRB1	9.39	6.08	13.1	0	19.13
HLA‐DQB1	11.11	4.76	15.18	0	19.2
HLA‐DPB1	4.01	0.64	6.32	0	10.56
HED_D‐>R_					
HLA class I	5.74	4.59	7	0	9.76
HLA‐A	6.12	3.36	8.4	0	10.88
HLA‐B	7.45	5.86	9.04	0	13.09
HLA‐C	4.77	3.14	5.87	0	7.54
HLA class II	6.7	4.15	8.43	0	13.58
HLA‐DRB1	8.28	4.12	10.5	0	17.1
HLA‐DQB1	9.02	0	13.72	0	18.53
HLA‐DPB1	3.79	0	6.14	0	10.56

*Note:* HED values are calculated as the genetic distance between allele pairs at each HLA locus in the recipient (HED_R_), donor (HED_D_) and mismatched haplotypes (HED_MM_) using the Grantham distance. HED_D‐>R_ is calculated as defined in Figure 1.

**TABLE 3:** Impact of HED on HSCT outcomes.

Variable	OS		DFS		NRM		CIR		aGVHD	
	HR	*p*	HR	*p*	HR	*p*	HR	*p*	HR	*p*
HED_R_										
HLA class 1	2.325	0.019	1.943	0.051	1.58	0.44	1.9	0.12	0.94	0.85
HLA‐A	1.76	0.117	1.945	0.042	1.1	0.87	2.24	0.038	0.55	0.054
HLA‐B	1.023	0.951	0.916	0.801	1.06	0.92	0.88	0.76	1.45	0.18
HLA‐C	2.025	0.047	1.723	0.091	3.64	0.035	1.05	0.89	1.12	0.67
HLA class 2	0.86	0.704	0.905	0.78	1.8	0.31	0.6	0.28	1.12	0.7
HLA‐DRB1	0.752	0.457	0.754	0.416	0.93	0.91	0.71	0.41	1.34	0.29
HLA‐DQB1	0.869	0.71	0.903	0.765	1.28	0.67	0.75	0.49	0.91	0.73
HLA‐DPB1	0.985	0.966	0.892	0.736	2.23	0.17	0.52	0.15	1.34	0.29
HED_D_										
HLA class 1	0.778	0.541	0.749	0.449	1.32	0.65	0.53	0.22	1.01	0.97
HLA‐A	0.481	0.074	0.582	0.13	0.58	0.41	0.64	0.29	1.08	0.79
HLA‐B	1.057	0.892	1.103	0.79	1.09	0.89	1.1	0.82	0.63	0.17
HLA‐C	1.504	0.265	1.725	0.098	0.79	0.72	2.23	0.038	0.81	0.47
HLA class 2	0.93	0.866	0.878	0.743	1.12	0.87	0.8	0.64	1.15	0.67
HLA‐DRB1	0.855	0.73	0.809	0.612	0.69	0.63	0.91	0.84	1.31	0.39
HLA‐DQB1	1.016	0.968	0.927	0.829	1.95	0.24	0.58	0.24	1.11	0.72
HLA‐DPB1	1.405	0.346	1.025	0.941	2.08	0.22	0.67	0.34	0.72	0.25
HED_MM_										
HLA class 1	1.254	0.58	1.133	0.754	0.86	0.84	1.26	0.63	1.06	0.87
HLA‐A	0.923	0.835	0.84	0.626	1.13	0.83	0.74	0.51	1.07	0.81
HLA‐B	0.624	0.298	0.482	0.1	0.9	0.88	0.34	0.092	0.79	0.46
HLA‐C	1.085	0.821	1.194	0.586	0.79	0.71	1.39	0.4	0.78	0.36
HLA class 2	0.64	0.297	0.688	0.325	0.49	0.34	0.86	0.74	0.68	0.19
HLA‐DRB1	0.801	0.587	0.886	0.734	1.09	0.88	0.81	0.64	0.66	0.17
HLA‐DQB1	0.831	0.629	0.808	0.532	0.99	0.99	0.76	0.51	0.71	0.21
HLA‐DPB1	0.978	0.953	1.052	0.879	0.74	0.64	1.21	0.63	0.82	0.48
HED_D‐>R_										
HLA class 1	1.674	0.159	1.363	0.373	0.94	0.92	1.58	0.27	0.89	0.7
HLA‐A	2.122	0.04	2.09	0.028	0.99	0.99	2.57	0.018	0.9	0.74
HLA‐B	0.445	0.097	0.363	0.034	0.26	0.18	0.48	0.16	1.1	0.76
HLA‐C	0.897	0.79	1.015	0.968	0.57	0.47	1.31	0.53	0.7	0.24
HLA class 2	0.653	0.346	0.816	0.592	0.97	0.96	0.78	0.6	0.62	0.14
HLA‐DRB1	0.699	0.403	0.678	0.327	0.56	0.44	0.8	0.63	0.99	0.98
HLA‐DQB1	0.939	0.877	1.077	0.836	1.32	0.64	0.94	0.9	0.58	0.093
HLA‐DPB1	0.699	0.383	0.593	0.172	1.48	0.5	0.33	0.043	1.26	0.4

*Note:* Impact of per‐class and per‐locus divergence variables on overall survival (OS), disease‐free survival (DFS), non‐relapse mortality (NRM), cumulative incidence of relapse (CIR) and acute GVHD (aGVHD). Unadjusted Hazard ratios (HR) and *p*‐values of HEDs categorised according to the lower quartile are based on univariate Cox model.

We next analysed donor HLA molecules and found no significant impact of either HED_D_ or HED_MM_ at any HLA locus on haplo‐HSCT outcomes (Table 3), precluding their use as a tool to help choose the best haploidentical donor.

**FIGURE 3:**
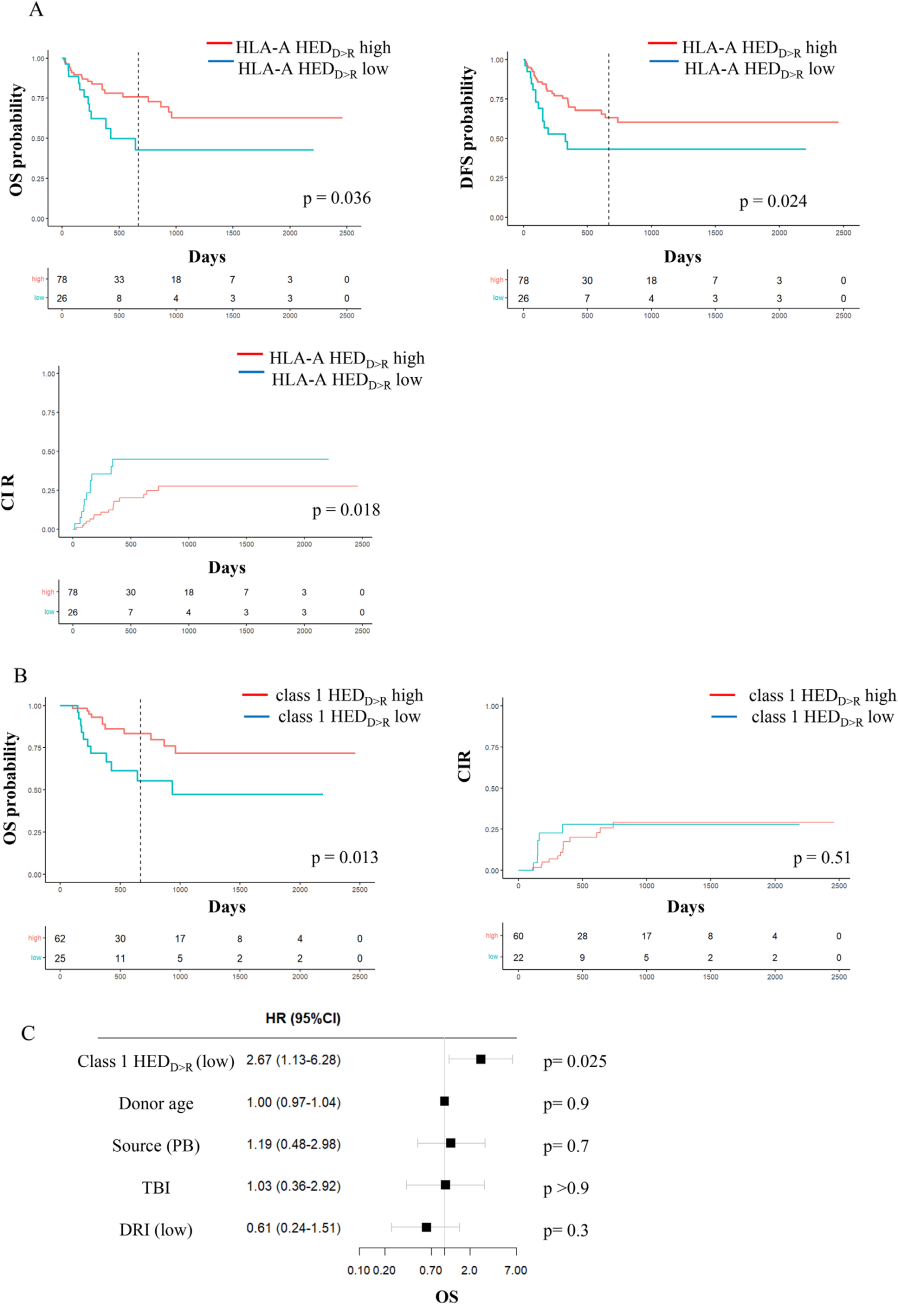
Survival estimates based on HED_D‐>R_ values. (A) Univariable Kaplan Meier estimates of probability of overall survival (OS), disease‐free survival (DFS) and cumulative incidence of relapse (CIR) based on HLA‐A HED_D‐>R_ values above or below the first quartile. Non adjusted *p*‐values indicate the significance of the log‐rank test. Vertical dotted line indicates the median follow‐up time. (B) Probability of OS and relapse based on class 1 HED_D‐>R_ values above or below the first quartile, excluding patients with < 100 days of follow‐up. (C) Multivariable cox regression analysis of OS including class 1 HED_D‐>R_ as main effect variable. Hazard ratios (HR) and *p*‐values are indicated.

We therefore explored a novel, tailored metric (HED_D‐>R_) that could indirectly capture the diversity of the immunopeptidome presented by recipient HLA molecules but not shared with the donor, assuming it could allow for a quantitative assessment of donor versus recipient alloreactivity. The HED_D‐>R_ calculation tool is publicly available via our online calculator (https://abched.pythonanywhere.com). Representative examples with HLA typing data of the recipient and the donor are shown in Figure 1 and all HED_D‐>R_ values derived from HLA genotyping data in our cohort are provided (Data Source S1). Patients with low HED_D‐>R_ at the HLA‐A locus had reduced OS (*p* = 0.036) and DFS (*p* = 0.024) and higher incidence of relapse (*p* = 0.018) (Table 2 and Figure 3A). Moreover, a landmark analysis for patients surviving at least 100 days showed that low class 1 HED_D‐>R_ was associated with inferior OS (*p* = 0.013), with no impact on relapse (Figure 3B). This effect on OS remained significant in multivariate analysis including the same covariables as above (HR 2.67, 95% CI 1.13–6.28; *p* = 0.025) (Figure 3C).

## 4 Discussion

The successful implementation of PTCy as a GVHD prophylaxis regimen has markedly improved outcomes of haplo‐HSCT, with survival rates similar to those after HLA‐matched HSCT under conventional GVHD prophylaxis. However, since several potential haploidentical donors are most often available within the patient's family, the selection of the optimal donor is a frequent issue in clinical practice. Certain HLA characteristics, including a B‐leader match, an HLA‐DRB1 mismatch in the graft‐versus‐host direction and an HLA‐DPB1 nonpermissive mismatch, are associated with improved survival and might be considered when selecting the donor [2]. Non‐permissive HLA mismatches are associated with excessive T‐cell alloreactivity, consistent with the observation that limited T‐cell alloreactivity is sufficient for GVL effect without inducing severe GVHD. The frequency and diversity of alloreactive T‐cell clonotypes has been shown to depend on the degree of immunopeptidome overlaps between the mismatched HLA alleles (predicted by PBM similarities), which itself reflects the genetic divergence in the antigen‐binding groove [6, 15, 19].

Using the HED_MM_ metric, we therefore analysed the divergence between the recipient/donor mismatched haplotype, but found no impact on haplo‐HSCT outcomes, in contrast to the recently reported association of higher HED_MM_ with increased risk of relapse [20, 21]. While this discrepancy may be related to the different HED calculation methods used in these studies, we reasoned that the HED_MM_ metric, which does not account for the HLA molecules from the shared haplotype, fails to capture a portion of the immunopeptidome that is common between the donor and the recipient and therefore does not contribute to T‐cell alloreactivity. Consequently, we developed a new HED_D‐>R_ metric that estimates the fraction of the recipient's immunopeptidome that is not recognised as self by the donor and found that a higher class 1 HED_D‐>R_ could inform about the probability of a better survival and lower risk of relapse. We propose that HED_D‐>R_ might affect the diversity of post‐transplant T‐cell specificities, and could therefore represent an immunogenetic predictor possibly influencing the GVL effect after haplo‐HSCT. Whether higher HED_D‐>R_ is indeed associated with increased diversity of alloreactive T‐cell clonotypes remains to be confirmed by deep T cell receptor (TCR) sequencing.

Of note, the impact of class‐1 HED_D‐>R_ on survival became evident only among patients who survived beyond 100 days. This supports the hypothesis that the alloreactive effect captured by the HED_D‐>R_ metric may emerge over time, once the influence of intrinsically aggressive diseases (beyond immunological control) has diminished. It is also possible that opposing effects of HED_D‐>R_ on aGVHD and relapse in the early post‐transplant period may compensate for each other, leading to a lack of net impact on survival when patients who died prematurely are included. However, we were unable to formally demonstrate this phenomenon in our study. Importantly, the effect of HED_D‐>R_ on survival was observed despite the PTCy‐based regimen used for GVHD prophylaxis in all patients, providing further evidence to the recent observation that the use of PTCy does not mitigate the impact of HLA differences between donor and recipient [17]. Nevertheless, it cannot be excluded that PTCy may have accounted for the lack of detectable effect of HED_D‐>R_ on the incidence of aGVHD.

Our study is limited by its single centre and retrospective nature, and by the relatively small number of patients which may explain the lack of impact of disease‐ or transplant‐related characteristic on HSCT outcomes. For the same reason, the lack of a significant effect of class 1 HED_R_ on relapse despite its effect on survival may be due to low statistical power. However, if confirmed in larger patient cohorts, our observations may have important practical implications, as our freely available HED_D‐>R_ tool could help select the most appropriate donor among haploidentical candidates. Finally, our findings suggest new strategies for the selection of not only potential haploidentical donors but also mismatched unrelated donors (UDs). Indeed, introduction of PTCy‐based GVHD prophylaxis has also decreased the clinical risks associated with HLA mismatches in UDs, and mismatched UDs nowadays represent a promising option to improve access to transplant for patients without a fully matched donor. Class 1 HLA mismatches, especially at the antigen and PBM level, are associated with inferior survival in contemporary unrelated HSCT, even in PTCy transplants [17]. Whether HED_D‐>R_ could also inform about the probability of a better survival and lower risk of relapse in the unrelated HSCT setting is currently under investigation.

## Author Contributions

D.J.C., J.V. and V.A. conceived the study and analysed data. A.X., O.F., L.T. and R.P.L. provided clinical or biological data. B.A.L. performed statistical analyses. D.J.C., V.A. and S.C.‐Z. wrote the manuscript.

## Funding

The authors have nothing to report.

## Ethics Statement

All patients gave their consent for data collection before transplantation. The study did not require institutional review board (IRB) approval.

## Conflicts of Interest

The authors declare no conflicts of interest.

## Supporting information


**Data S1:** tan70491‐sup‐0001‐DataS1.xlsx.

**Figure S1:** Distribution of HED values in the recipients.

## Data Availability

The data that support the findings of this study are available in the Supporting Information of this article.
