# Supplementary material for: Harnessing HLA Divergence for Improved Donor Selection in Haploidentical Haematopoietic Stem Cell Transplantation
Source: HLA. 2025 Dec 10;106(6):e70491. doi: 10.1111/tan.70491 (PMC12691898; doi:10.1111/tan.70491)
Supplement: Supplementary file 2 — Figure S1: Distribution of HED values in the recipients. [file TAN-106-e70491-s002.pdf]

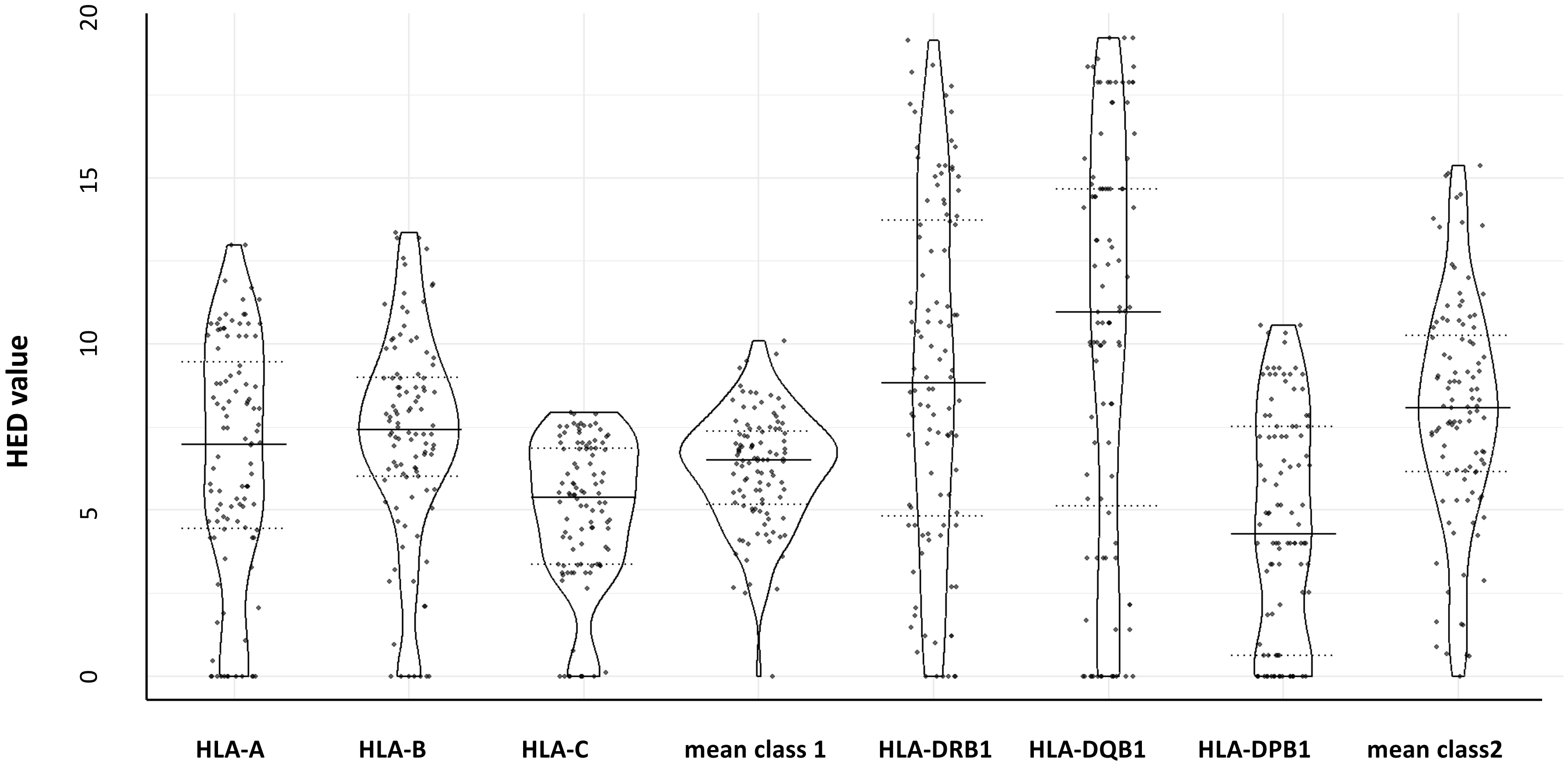

Supplementary Figure 1: Distribution of HED values in the recipients.

Each plots represents the HED value of one patient at the indicated locus. Horizontal bars indicate the median value and the 25th and 75th percentiles (dotted lines).
